# Investigation of the Suzuki–Miyaura cross-coupling reaction on a palladium H-beta zeolite with DFT calculations

**DOI:** 10.1038/s41598-023-51116-x

**Published:** 2024-01-05

**Authors:** Bundet Boekfa, Thana Maihom, Masahiro Ehara, Jumras Limtrakul

**Affiliations:** 1 Division of Chemistry, Department of Physical and Material Sciences, Faculty of Liberal Arts and Science, Kasetsart University, Kamphaeng Saen Campus, Nakhon Pathom, 73140 Thailand; 2 Center for Advanced Studies in Nanotechnology for Chemical, Food and Agricultural Industries, Kasetsart University Institute for Advanced Studies, Kasetsart University, Bangkok, 10900 Thailand; 3 Institute for Molecular Science, Nishigo-naka 38, Myodaiji, Okazaki, 444-8585 Japan; 4 Department of Materials Science and Engineering, School of Molecular Science and Engineering, Vidyasirimedhi Institute of Science and Technology, Rayong, 21210 Thailand

**Keywords:** Catalytic mechanisms, Heterogeneous catalysis, Electronic structure

## Abstract

Metal or metal cluster-doped zeolites catalyse a wide variety of reactions. In this work, a coupling reaction between bromobenzene and phenylboronic acid to yield biphenyl with the Pd–H-Beta zeolite catalyst was investigated with density functional theory (DFT) calculations. Utilizing a model system with tetrahedral Pd_4_ clusters within the H-Beta zeolite, it was demonstrated that the catalyst exhibited notable reactivity by effectively reducing the activation energy barrier for the reaction. Our investigation revealed that the zeolite framework facilitated electron transfer to the Pd cluster, thereby increasing the reaction activity. The coupling reaction was shown to be exothermic and comprise three main steps: oxidative addition of bromobenzene (C_6_H_5_Br), transmetallation with phenylboronic acid (C_6_H_5_B(OH)_2_), and reductive elimination of biphenyl (C_12_H_10_). Specifically, in the transmetallation step, which was the rate-determining step, the C–B bond breaking in phenylboronic acid (C_6_H_5_B(OH)_2_) and the phenylboronate anion (C_6_H_5_B(OH)_3_^–^) were compared under neutral and basic conditions, respectively. This comprehensive study clarifies the mechanism for the reaction with the modified Pd zeolite catalyst and highlights the essential role of the zeolite framework.

## Introduction

Suzuki–Miyaura cross-coupling is a reliable approach for organic synthesis, and it is a highly selective method for creating C–C bonds with high yields under mild conditions^1,2^. It was originally developed with homogeneous catalysts by Heck, Negishi and Suzuki et al.^1,3^, and various modifications of the reaction have been reported since then. Recently, reactions producing high-value molecules have been achieved with heterogeneous catalysts such as anchored nanoparticles on metal oxides and nanoparticle dispersions in liquid media^4–8^. For example, biaryl derivatives can be produced from aryl halides and aryl boronic acid with Au/Pd alloy nanoparticles or Pd-zeolite catalysts^5,8,9^.

The use of a palladium zeolite catalyst has been explored for cross-coupling reactions to synthesize unsymmetric biaryls from aryl halides and aryl boronic acids^9–12^. This catalyst holds significant promise for syntheses of fine chemicals in pharmaceuticals and agrochemicals fields^13^. The capabilities of these catalysts have been assessed with specific zeolites such as FAU^9,11^, BEA^12^ and ZSM-5^10,14^. Notably, the Pd siliceous ZSM-5 zeolite catalysed the Suzuki–Miyaura cross-coupling reaction with a low Pd loading under ambient conditions^10^. By employing K_2_CO_3_ in ethanol as the base with this catalyst, the coupling reaction of bromobenzene and phenylboronic acid yielded biphenyl with a high yield of 96%. Furthermore, Pd-zeolites have been utilized for NOx decomposition^15^ and methane oxidation^16–19^. Among the various Pd-zeolite catalysts, Pd-Beta zeolite exhibits notable shape selectivity for coupling reactions due to its large specific pore sizes^12,20^. The Pd-Beta zeolite shows superior catalytic activity and recyclability in comparison to other catalysts. Its larger pores allow efficient encapsulation of aggregated Pd species, thereby enhancing the catalytic performance. Additionally, the active Pd species are effectively immobilized by the negative charges in the zeolite framework, which also contributes to the enhanced activity and stability.

However, theoretical investigations of Pd zeolite heterogeneous catalysts have been limited thus far. Moc et al. conducted density functional theory (DFT) calculations on a Pd_4_ cluster within a zeolite to examine dissociation of the H_2_ molecule, and specifically utilized a siliceous FAU zeolite as the support^21^. In their work, the Pd_4_ cluster was found to be stable in the triplet state of the tetrahedral (T_d_) structure. The reverse-hydrogen spillover process was also investigated with the Pd_4_ cluster on the H-FAU zeolite^22^. A hybrid quantum–mechanical/molecular-mechanics (QM/MM) method was used to study the Au and Au–Pd clusters with T_d_ structures within the TS-1 zeolite^23^. Furthermore, periodic DFT calculations using Pd clusters with various sizes in MOR zeolite were performed to examine the adsorption of NO^24^. However, theoretical studies on the coupling reactions catalysed with Pd-zeolites remain limited because of the complexity and large computational cost. Zeolites are widely employed as cost-effective and environmentally friendly catalysts, and investigating the coupling reactions within Pd zeolite catalysts holds significant potential to enhance our understanding of the reaction mechanisms and enable catalyst improvements.

Derouane proposed the concept of the zeolite framework effect or confinement effect for the adsorption and reactions inside the zeolite^25^. However, accurately representing the confinement effect in DFT calculations requires careful selection of the functionals. Standard hybrid DFT functionals are inadequate for describing the confinement effects, so the M06 series of functionals was adopted to study substrate adsorption and reactions in zeolite frameworks^26–28^. The M06 functional group provides high-quality results, as demonstrated in numerous previous studies on hydrocarbon adsorption and the reaction mechanisms of zeolites^27,29–36^. In particular, the M06-L functional^26^ has been used to investigate the mechanisms of reactions occurring on gold-^30,32^, iron-^29^ and vanadium-modified^31^ zeolites. Additionally, it has been used for different zeolites with various pore sizes, such as FER, ZSM-5, FAU, and MCM-22, which are used in methane activation^32^. The M06 functional was used to study the synthesis of coumarin with the large pore Beta zeolite^33^. More recently, the M06-L functional was used to study the conversion of ethanol to acetaldehyde over zinc Beta Zeolite^37^. These functionals effectively described the confinement effects of the zeolites, and the theoretical studies described the differences among zeolites with various pore sizes and stabilization of the transition states for metal-zeolites. By comparing the experimental results with the theoretical calculations, valuable insights into the reaction mechanisms were obtained.

In this study, DFT calculations and the M06-L functional were used to investigate the Suzuki–Miyaura coupling reaction between bromobenzene and phenylboronic acid over Pd–H-Beta zeolite. Our primary focus in this study was on a theoretical exploration of this reaction with heterogeneous Pd catalysts supported on H-Beta zeolite and the influence of the base. This study represents the first comprehensive investigation of the mechanisms for coupling reactions involving Pd–H-Beta zeolite catalysts. To simulate the coupling reaction and represent the Pd–H-Beta zeolite, we utilized the Pd_4_ cluster with the 34T system^33^, where T denotes tetrahedral coordination of the Si or Al atoms. The results of our study demonstrate that the cross-coupling reaction proceeds similarly to those with standard homogeneous Pd catalysts^6^. This research provides a comprehensive understanding of the reaction mechanisms and energetics of heterogeneous Pd zeolite catalysts.

## Results and discussion

### Structure of Pd_4_–H-beta zeolite

**Figure 1:**
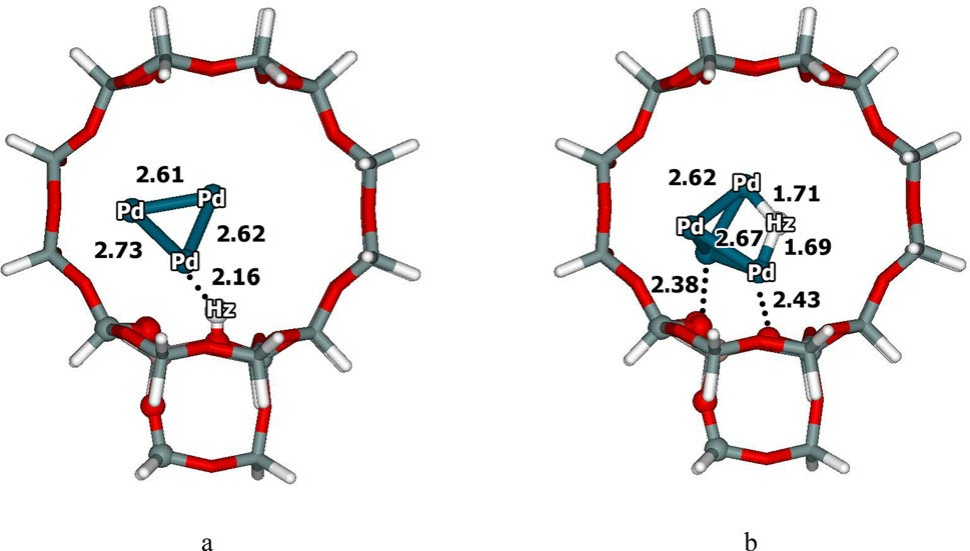
Structures of the Pd_4_-H-Beta zeolite Model 34T quantum cluster optimized by the M06-L/6-31G(d,p) + LANL2DZ calculations (distances are in Å).

First, the stable structures and coordination modes of the Pd_4_ clusters within the H-Beta zeolite were explored by optimizing various initial structures. The isolated Pd cluster had a stable structure and was a triplet-state tetrahedral Pd_4_ cluster. The clusters were predominantly located above the Brønsted acid sites in the 12T straight channels of the beta zeolite, where the coupling reactions occur. As shown by a literature review, the Pd_4_ clusters inside zeolites are tetrahedral clusters^21,23,24^. Two modes for Pd_4_ cluster adsorption on the H-Beta zeolite were identified. The first involved adsorption of the Pd_4_ clusters over the Brønsted acid sites, as depicted in Fig. 1a. The second type involved complete proton transfer from the Brønsted acid to the Pd_4_ cluster, as shown in Fig. 1b. The corresponding energies for adsorption of the Pd_4_ clusters on H-Beta zeolite were − 152.3 and − 181.1 kcal mol^-1^, respectively. The latter structure was more stable and exhibited higher activity for the reaction.

The calculated structure of the Pd_4_–H-Beta zeolite was analysed. The calculated average Al–O bond distance of the 34T Pd_4_–H-Beta zeolite framework was within the range of 1.69–1.74 Å. The Pd_4_ cluster was slightly deformed, and the Pd–Pd bond distances were 2.62–2.67 Å. Notably, the proton from the zeolite was transferred to the Pd_4_ cluster, resulting in Pd–H bond distances of 1.69 and 1.71 Å. Furthermore, the Pd cluster interacted with the zeolite framework, and the Pd⋅⋅⋅O bond distances ranged from 2.38 to 2.64 Å. These values were consistent the extended X-ray absorption fine structure (EXAFS) analysis; the Pd⋅⋅⋅O distances were within the range of 2.74–2.76 Å for small Pd clusters on Na-X FAU zeolite^38^.

Charge transfer from three bridging oxygen atoms of the zeolite framework to the Pd cluster was also observed, resulting in an increase in the electron density of the Pd_4_ cluster. The Mulliken charge of the Pd_4_ cluster inside the H-Beta zeolite was analysed. The charge transfer to the Pd_4_ cluster, which was approximately − 0.57 |e|, left a charge on the zeolite of + 0.57 |e|. Because of the confinement effect resulting from the presence of the 34T quantum cluster around the Pd cluster, the oxygen framework of the zeolite functioned as a Lewis base^39^ by transferring electrons to the Pd cluster. The anionic Pd cluster exhibited oxidation by lowering the activation barrier^40^. In addition, we examined the possible spin states of the Pd_4_-H-Beta system, including the singlet to quintet spin states. Among these, the most stable spin state was found to be the triplet state. These results agreed with previous calculations for the Pd_4_-FAU zeolite^21^.

### Coupling reaction of bromobenzene with phenylboronic acid on the Pd_4_–H-beta zeolite

**Figure 2:**
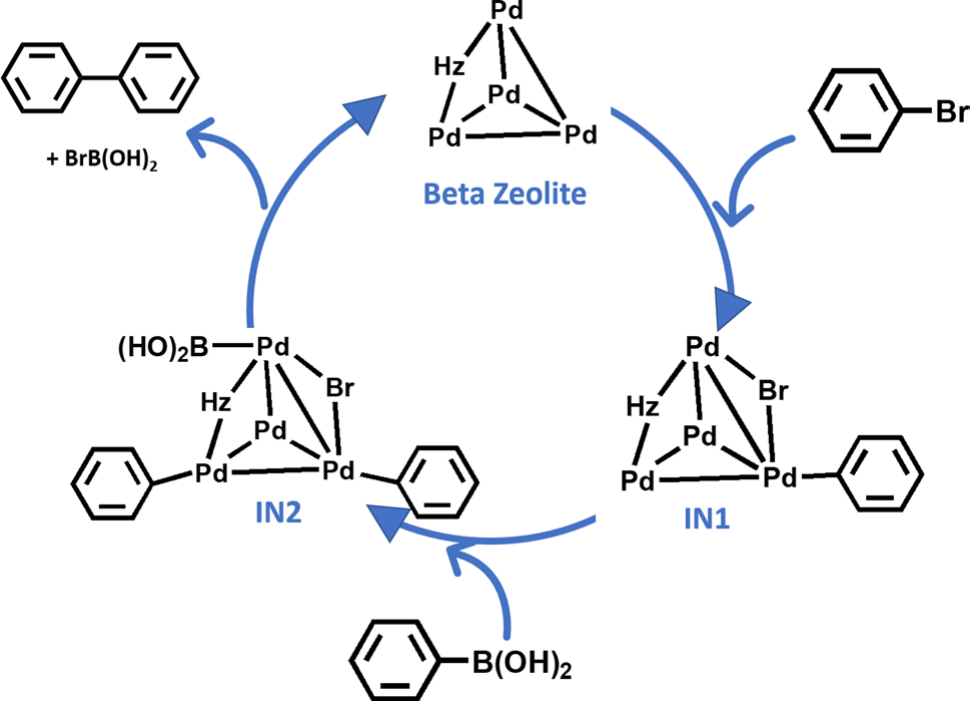
The catalytic cycle for coupling of bromobenzene and phenylboronic acid over the Pd–H-Beta zeolite.

**Figure 3:**
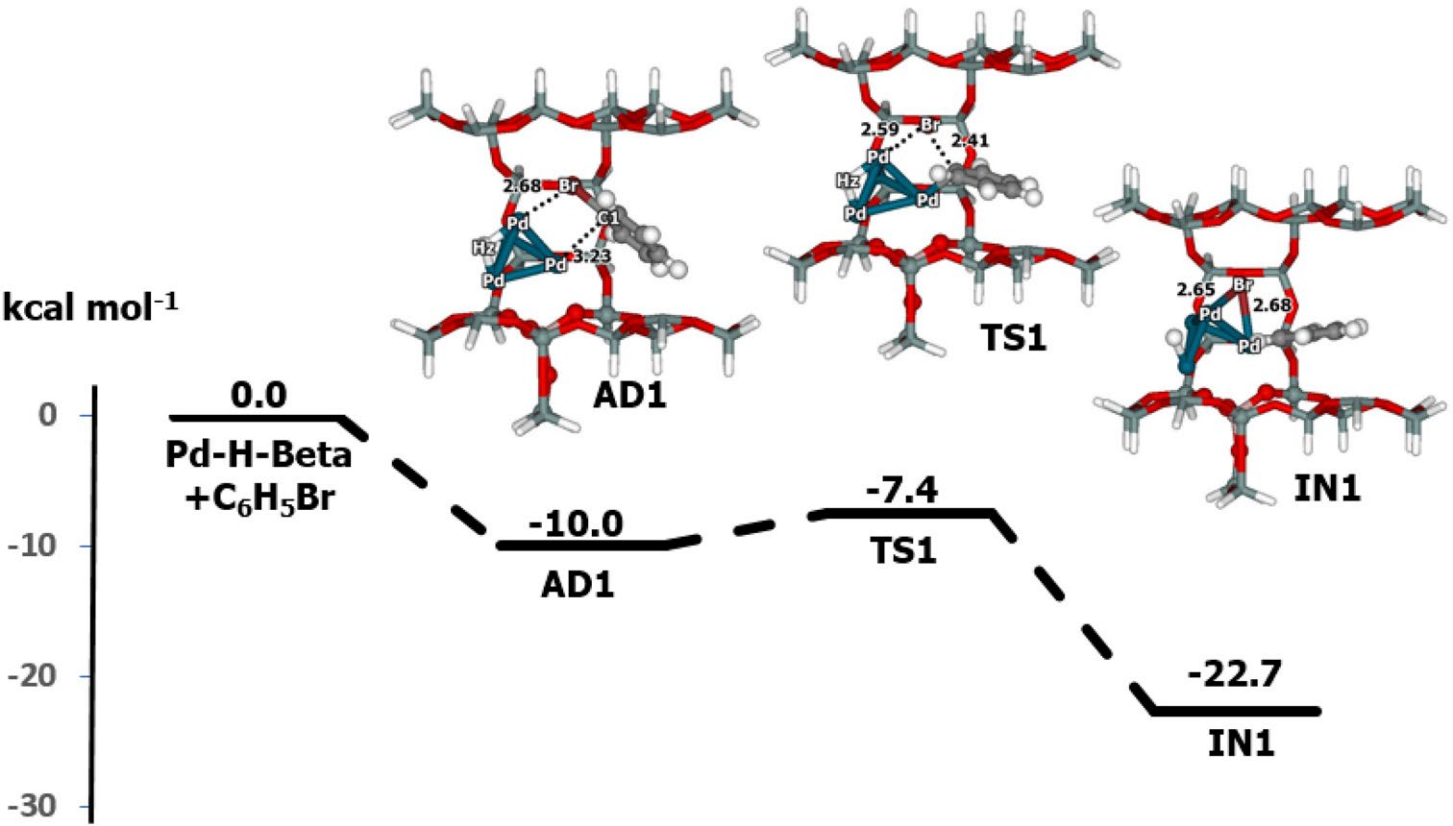
Energy profile for the oxidative addition of bromobenzene on Pd–H-Beta zeolite determined with M06-L/6-311G(d,p) + Lanl2DZ//M06-L/6-31G(d,p) + Lanl2DZ DFT calculations (distances are in Å).

**Figure 4:**
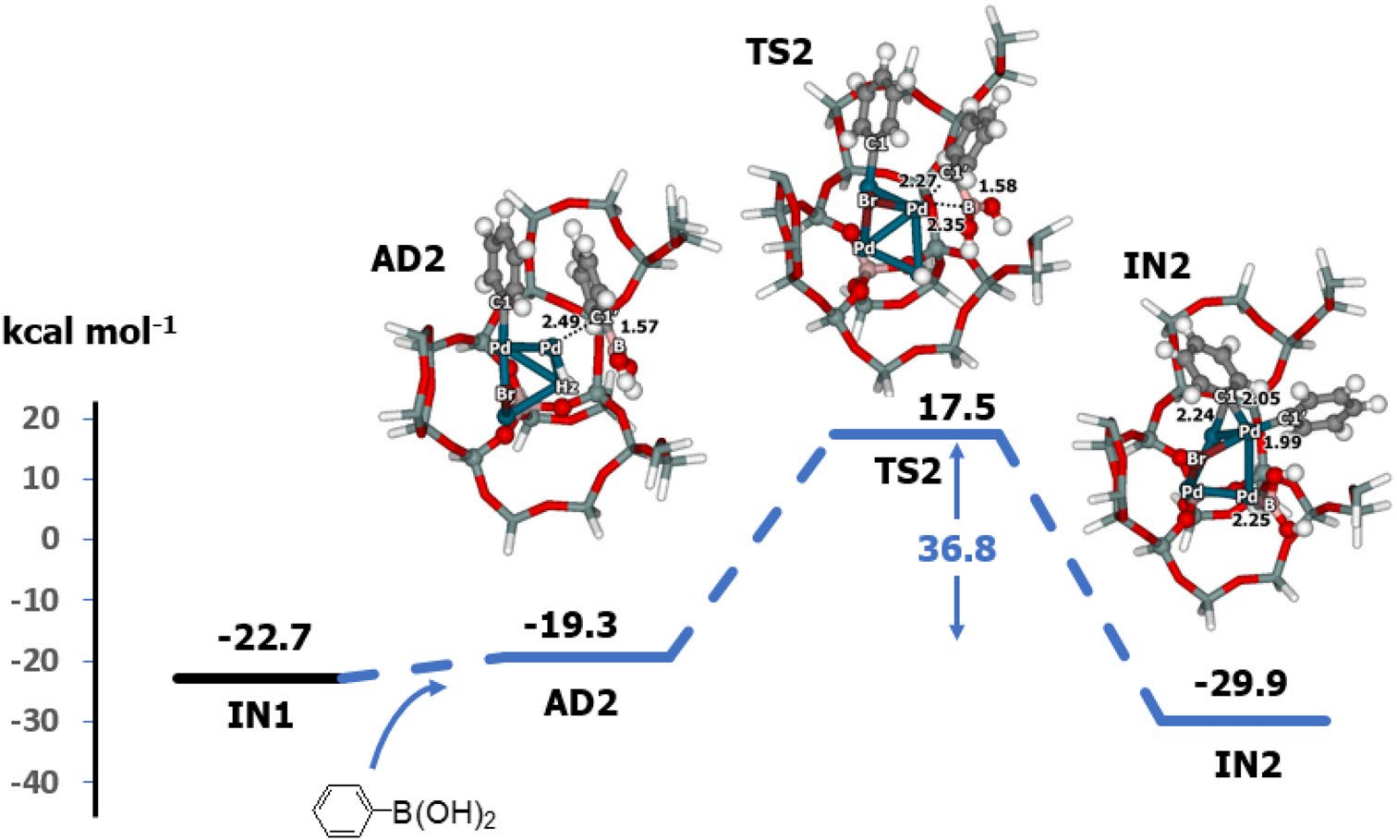
Energy profile for transmetalation with phenylboronic acid on the Pd–H-Beta zeolite, as determined with M06-L/6-311G(d,p) + Lanl2DZ//M06-L/6-31G(d,p) + Lanl2DZ DFT calculations (distances are in Å).

**Figure 5:**
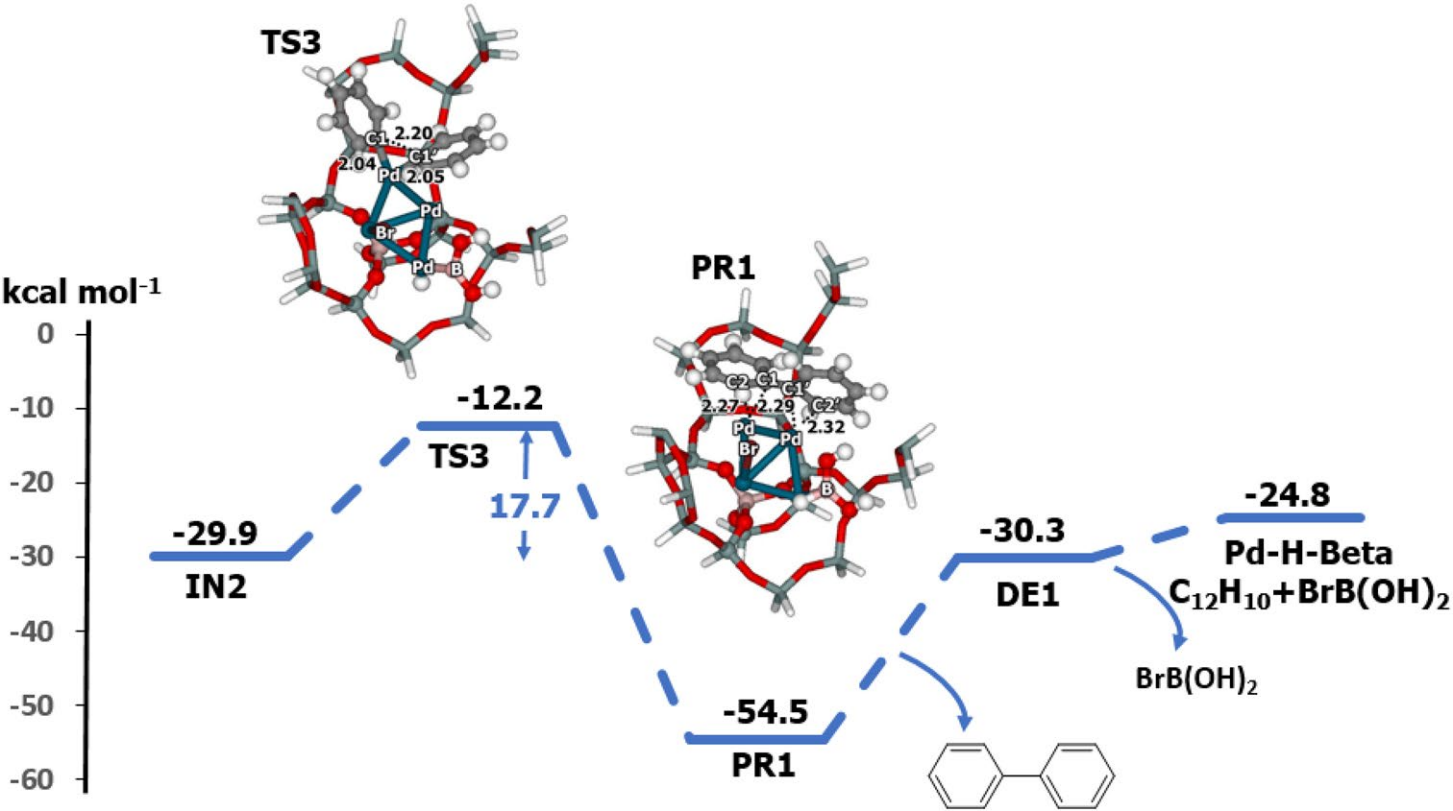
Energy profile for the reductive elimination of biphenyl on Pd–H-Beta zeolite, determined with M06-L/6-311G(d,p) + Lanl2DZ//M06-L/6-31G(d,p) + Lanl2DZ DFT calculations (distances are in Å).

The coupling of bromobenzene and phenylboronic acid on Pd_4_–H-Beta, which formed biphenyl as the major product, was investigated. The standard catalytic cycle for the coupling reaction was assumed under neutral conditions, and phenylboronic acid C_6_H_5_B(OH)_2_ was considered as the substrate. Additionally, we also considered the case for phenylboronate C_6_H_5_B(OH)_3_^–^ under basic conditions, as shown later. As depicted in Fig. 2, the reaction proceeded through a catalytic cycle consisting of three steps: oxidative addition of the bromobenzene, transmetalation with phenylboronic acid and reductive elimination of biphenyl. The calculated energy profiles are shown in Figs. 3, 4 and 5.

In the first step, oxidative addition, the C–Br of bromobenzene is broken, and C-Pd and Br-Pd bonds are formed with the Pd_4_–H-Beta zeolite. Initially, bromobenzene is adsorbed on the Pd_4_–H-Beta system (**AD1**), with the Br atom pointing towards Pd (Br-Pd distance is 2.68 Å). At **AD1**, the Pd–Pd bond length increases from 2.62 to 2.68 Å, while the other geometrical changes are minor; the C1–Br bond increases by only 0.06 Å. At this step, the Mulliken charge of the Pd_4_ cluster is changed to − 0.75 |e|, that of the zeolite framework is + 0.59 |e|, and that of C_6_H_5_Br is + 0.16 |e|. For the adsorption process, we found that C_6_H_5_Br acts as a Lewis basis by transferring electrons to the Pd_4_–H-Beta zeolite framework. Charge reorganization occurs from the Br atom to the Pd cluster, which increases the Coulomb interaction. The Gibbs free energy for adsorption of bromobenzene on Pd_4_–H-Beta was calculated as − 10.0 kcal mol^−1^.

Bromobenzene undergoes dissociative chemisorption on Pd_4_–H-Beta to form the intermediate (**IN1**) through oxidative addition. This step is exothermic and releases 12.7 kcal mol^-1^. At the transition state, **TS1**, the C1–Br bond breaks, while the Pd1-C and Pd2-Br bonds simultaneously form. The imaginary frequency was calculated to be 134*i* cm^−1^. In **TS1**, the C1–Br bond length increases from 1.97 to 2.41 Å, while the Pd1-C1 bond contracts to 2.03 Å. The Pd1–Pd2 bond increases to 2.64 Å. The activation energy calculated for this step was 2.6 kcal mol^−1^. Comparatively, the present Pd catalyst displays high activity in the oxidative addition step. For instance, the activation energies calculated with the M06 functional^41^ were 9.6 kcal mol^−1^ for bromobenzene on Pd(PPh_3_)_2_, 12.6 kcal mol^−1^ for chlorobenzene on Au_10_Pd_10_ cluster^7^, 44.6 kcal mol^−1^ for iodobenzene on Au_20_ with M05-2X calculation^42^, and 9.3 kcal mol^−1^ for Au_10_Pd_10_-4PVP catalysts^40^. In **IN1**, benzene is bonded to the Pd1 atom with a Pd1-C1 distance of 1.97 Å. The Br atom is bridged by two Pd atoms with distances of 2.65 and 2.68 Å. This intermediate has a relative energy of − 22.7 kcal mol^−1^. At this step, the Pd_4_ cluster can be oxidized, since the Mullikan charge of the Pd_4_ cluster is − 0.66 |e|, the charge for bromobenzene is − 0.01 |e| and that for the zeolite is + 0.67 |e|.

The next step of the reaction is transmetalation, which is initiated by the adsorption of phenylboronic acid followed by C–B bond breaking (Fig. 4). Phenylboronic acid is adsorbs on a different Pd site of the Pd_4_–H-Beta in the zeolite channel, and an interaction between the phenyl ring and the Pd cluster yields a Pd–C distance of 2.49 Å (**AD2**). The relative energy of **AD2** is − 19.3 kcal mol^−1^. The transition state (**TS2**) involves C–B bond breaking of phenylboronic acid to form the **IN2** intermediate. At **TS2**, C–B bond-breaking and formation of the Pd–C and Pd–B bonds constitute the reaction coordinate, with one imaginary frequency of 207*i* cm^−1^. The activation energy for this step is as large as 36.8 kcal mol^−1^. This step was found to be the rate determining step of the coupling reaction. In **IN2**, the C2-B bond is dissociated at the Pd3 atom, and C2 forms bonds with two Pd atoms to give bond distances of 2.05 and 2.24 Å. Although the relative energy of **IN2** is − 29.9 kcal mol^−1^, the subsequent step yielding biphenyl is highly exothermic.

The last step is C–C bond formation and elimination of the biphenyl (Fig. 5). The transition state for this step (**TS3**) corresponds to formation of the C–C bond with one imaginary frequency of 240*i* cm^−1^. At **TS3**, the C1–C2 bond length is 2.20 Å, and the Pd-C1 bond length increases from 1.99 to 2.05 Å. This step requires an activation energy of 17.7 kcal mol^-1^. The biphenyl product is adsorbed on the cluster through an interaction between the phenyl ring and the Pd cluster, with a C2-Pd bond distance of 2.42 Å. The relative energy of the product **PR1** is − 54.5 kcal mol^-1^. Biphenyl is desorbed from the Pd cluster with a desorption energy of 24.2 kcal mol^-1^. Transmetalation was found to be the rate-determining step of the coupling reaction. The present results are consistent with previous calculations in which this step of the reaction has a high activation energy with organoboronic acid and neutral conditions, but the reaction has a low energy barrier under basic conditions (OH^−^)^43^.

**Figure 6:**
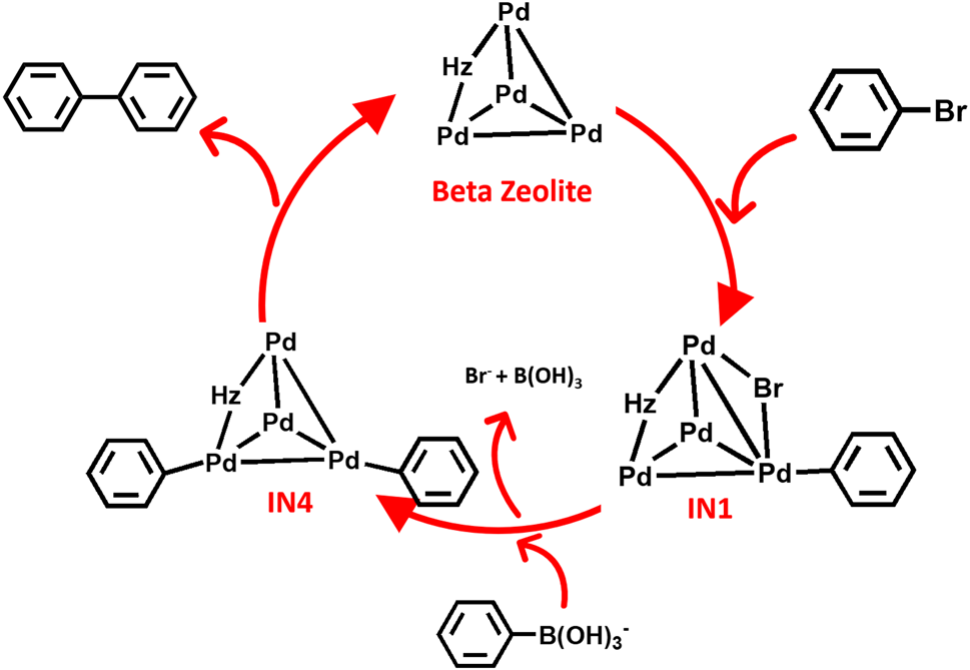
Catalytic cycle for the coupling reaction of bromobenzene and phenylboronate anion over Pd–H-Beta zeolite.

**Figure 7:**
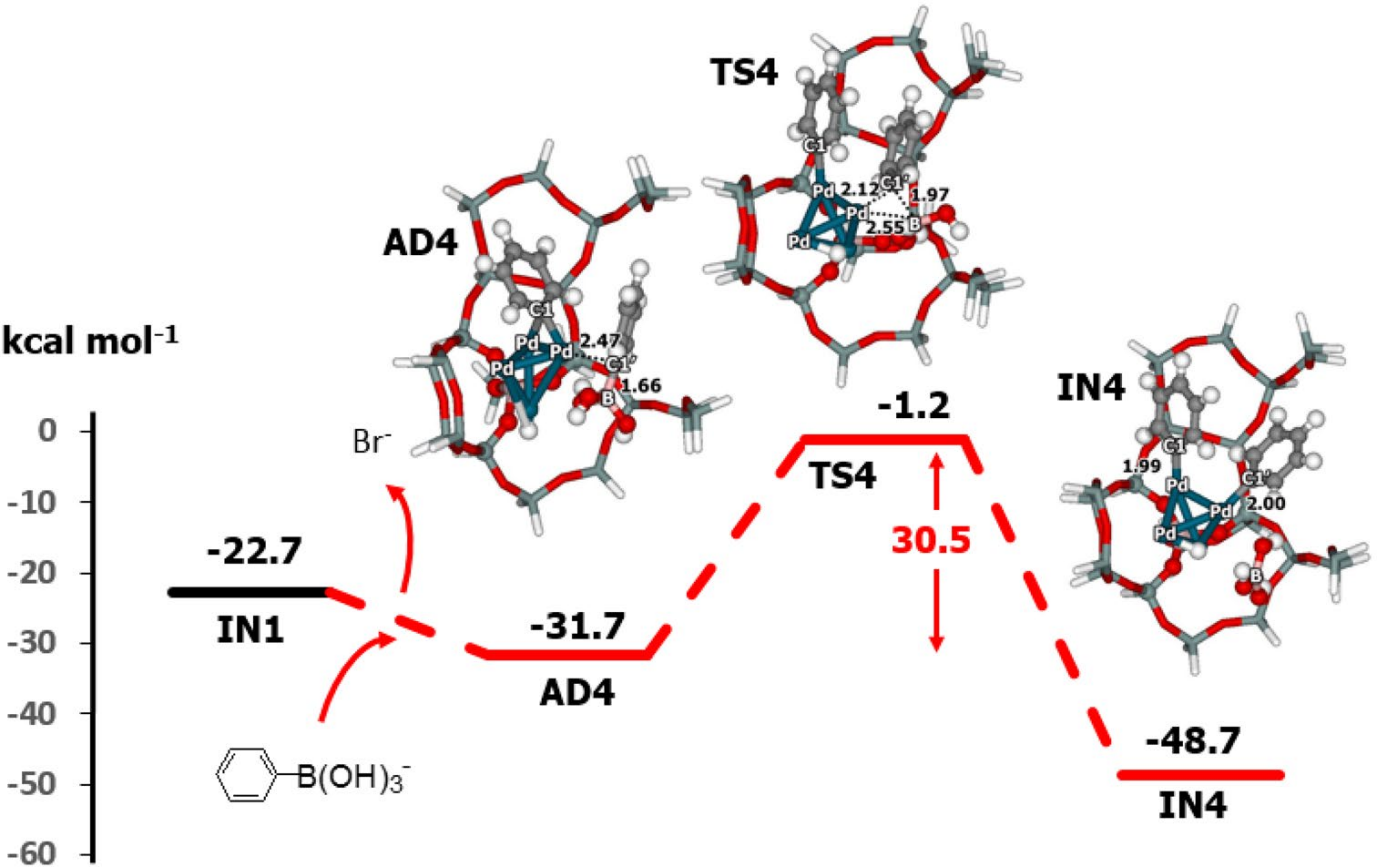
Energy profile for the transmetalation with phenylboronate anion on Pd–H-Beta zeolite, as determined with M06-L/6-311G(d,p) + Lanl2DZ//M06-L/6-31G(d,p) + Lanl2DZ DFT calculations (distances are in Å).

**Figure 8:**
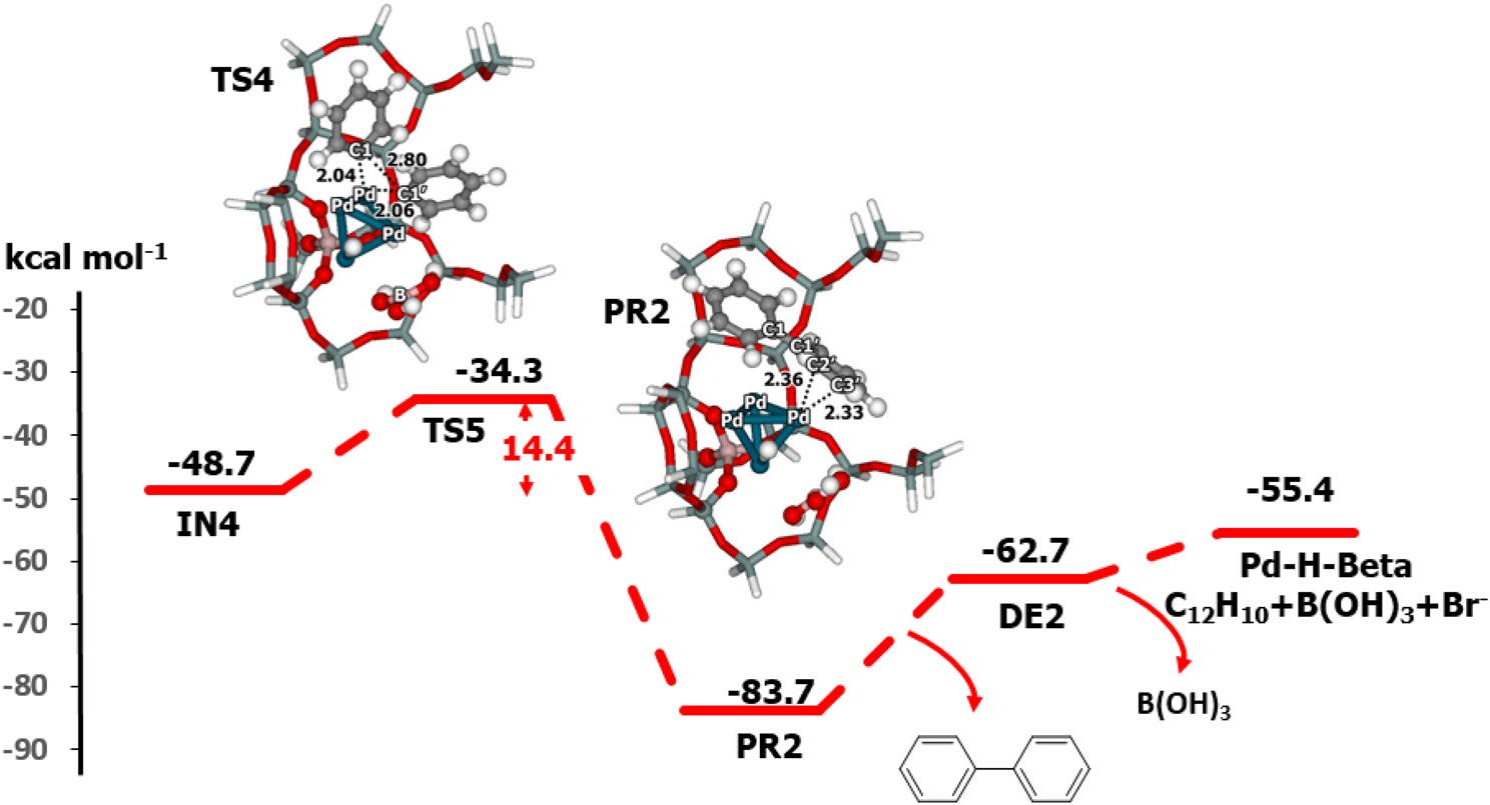
Energy profile for the reductive elimination of biphenyl from the Pd–H-Beta zeolite, as determined with M06-L/6-311G(d,p) + Lanl2DZ//M06-L/6-31G(d,p) + Lanl2DZ DFT calculations (distances are in Å).

The basic conditions may facilitate the reaction by forming the phenylboronate anion, C_6_H_5_B(OH)_3_^–^, which is more reactive and would reduce the activation barrier. Thus, the reaction mechanism operating under basic conditions was examined and is shown in Fig. 6. The energy profile for the basic system is shown in Figs. 7 and 8. In experiments with Pd-ZSM-5^10^, Pd-FAU^9,12^, Pd-Beta^12,20^ and Pd-catalysts^6,43^, it was shown that this step was accelerated by the base and solvent. The structures of the intermediates formed from C_6_H_5_B(OH)_3_^–^ are shown in Fig. 6. Adsorption of the phenylboronate anion with the loss of a bromide ion (Br^–^) gives the transient intermediate (**AD4**) with a relative energy of − 31.7 kcal mol^-1^. Then, the C–B bond breaks via the transition state (**TS4**) with an imaginary frequency at 212*i* cm^−1^. This step proceeds with an activation energy of 30.5 kcal mol^-1^. The activation barrier for the system with the phenylboronate anion is lower than that seen with phenylboronic acid by approximately 6.3 kcal mol^−1^. This step produces **IN4** with a relative energy of − 48.7 kcal mol^−1^. The reaction with the phenylboronate anion also produces the intermediate **IN4,** which is more stable than **IN2**. Under basic conditions, this step is exothermic by approximately 18.8 kcal mol^−1^. Boric acid (B(OH)_3_) can be desorbed from **IN4** to give product **PR2**. The desorption energy for boric acid is approximately 7.3 kcal mol^−1^.

For the phenylboronate anion pathway, the elimination of biphenyl occurs via **IN4** to give **PR2**. The structure of the transition state **TS4**, in which C–C bond formation occurs, is similar to that of **TS3**. One imaginary frequency at 153*i* cm^−1^ was found. This step requires an activation energy of 14.4 kcal mol^−1^. Biphenyl is the product of this step and has a relative energy of − 83.7 kcal mol^−1^. Biphenyl is desorbed from the Pd-zeolite cluster with a desorption energy of 21.0 kcal mol^−1^.

Biphenyl is the main product of the coupling reaction. During the reaction occurring under basic condition, the Br^–^ anion and B(OH)_2_^+^ cation are produced and form B_2_(OH)_4_, BrB(OH)_2_ and B(OH)_3_^8,43^. For example, two B(OH)_2_^+^ species from two phenylboronic acids (C_6_H_5_B(OH)_2_) form tetrahydroxydiborane B_2_(OH)_4_. In the phenylboronic acid pathway, the reaction produces BrB(OH)_2_ with a formation energy of − 24.8 kcal mol^−1^ relative to the isolated molecules or ions. Desorption of the BrB(OH)_2_ requires an energy of 5.5 kcal mol^−1^. At this step, the water solvent reacts with BrB(OH)_2_ to produce boric acid [B(OH)_3_] and HBr. The formation energy including the water (C_6_H_5_Br + C_6_H_5_B(OH)_2_ + H_2_O → C_12_H_10_ + B(OH)_3_ + HBr) is − 41.1 kcal mol^−1^. Water molecules are used in proceeding to the latter step to form boric acid. For the phenylboronate anion pathway, the reaction produces boric acid B(OH)_3_ and the Br^−^ anion with a formation energy of approximately − 55.4 kcal mol^−1^.

Differences in the energetics for the phenylboronic acid and phenylboronate anion transmetallation steps are clearly seen in Figs. 4 and 7. In the case with basic condition, the activation energy barriers after **IN2** (30.5 and 14.4 kcal mol^−1^) are lower than those of the neutral case (36.8 and 17.7 kcal mol^−1^). Additionally, the product **PR2** is more stable than the neutral product **PR1**. This means that the base facilitates the coupling reaction both kinetically and thermodynamically. We found that the supported zeolite framework has a significant impact on the activity of the Pd_4_ cluster. This is consistent with our previous work describing N_2_O decomposition^30^ and methane activation^32^ on Au-supported zeolites, which showed lower activation energies with the zeolite framework compared to those of the bare metal system. Moreover, the supported zeolite framework also provides the electron and converts the Pd_4_ cluster into an anionic species, which is found to catalyse the reaction^44,45^.

## Summary

The coupling of bromobenzene and phenylboronic acid on Pd–H-Beta zeolite was investigated with DFT calculations using the M06-L functional. The Pd_4_–34T–H-Beta model system was used to simulate the coupling reaction. In the Pd–H-Beta model, the bridged oxygen atoms donate electron density to activate the Pd cluster for the adsorption and coupling reactions. The mechanism for the coupling reaction consists of three steps: oxidative addition, transmetalation and reductive elimination. Oxidative addition of bromobenzene occurs at the active palladium site, which forms the intermediate with an activation barrier of 2.6 kcal mol^−1^. The second step, in which breaking of the C–B bond in phenylboronic acid occurs, was found to be the rate-determining step with an activation energy of 36.8 kcal mol^−1^. The activation energy for reductive elimination is approximately 17.7 kcal mol^−1^. The effect of the base was also studied with the phenylboronate anion. We found that the base facilitates a reaction, in which the calculated activation energies are only 30.5 and 14.4 kcal mol^−1^ for the second and third steps, respectively. The cross-coupling reaction proceeds exothermically with the production of biphenyl. We simulated the confinement effects of the zeolite framework and the impacts on the adsorption and the coupling reactions on the Pd–H-Beta zeolite and showed how the base facilitated the reaction.

## Methodology

The Pd–H-Beta zeolite catalyst was utilized to investigate the coupling reaction so as to take advantage of its ion-exchange capability, thermal stability, and size and shape selectivity. The effectiveness of the Beta zeolite in various hydrocarbon reactions is widely acknowledged, including the Pechmann condensation^33^, dimethylnaphthalene isomerisation^46^, and coupling reactions^12,20,47–51^. To model the Pd–H-Beta zeolite, a 34T cluster model, with T representing tetrahedral coordination of the Si or Al atoms, was generated from the Beta lattice structure^52^, including the intersections of the straight channels, as depicted in Fig. 1. Previous studies have shown that this model predicts the adsorption and activation energies of hydrocarbons while demonstrating good agreement with the experimental values^33,37,53^. Various configurations and locations of the Pd_4_ cluster within the zeolite pores were tested, and the 12T channel over a Brønsted acid site was identified as the preferred location for the Pd_4_ cluster.

The DFT method and the M06-L functional was used to calculate the structures and the energy profiles of the coupling reaction on the 34T quantum cluster of the Pd–H-Beta zeolite. The M06 family of functionals, including the M06-L method developed by Zhao and Truhlar, is known to be effective in studying various properties, such as thermochemistry, kinetics, noncovalent interactions, excited states, and transition properties^26,28^. Moreover, the M06-L method accurately represents the effects of the zeolite framework^27,28^. In the DFT calculations, the 6-31G(d,p) basis set was used for the Al, Si, O, C and H atoms, while the relativistic effective core potential (RECP) with the LANL2DZ basis set was adopted for the Pd and Br atoms. To contain the zeolite framework during geometry optimization, only the active regions [Si_3_O_3_Al(OH)Si] and probe molecules were allowed to relax, while the rest were kept fixed at the crystallographic structure^52^. The 34T Model H-Beta zeolite^33^ and H-ZSM-5 zeolite^27^ were studied with the M06 functional and provided adsorption and activation energies that compared well with experimental data. The transition states were confirmed through normal mode analyses, which revealed one imaginary frequency mode corresponding to the reaction coordinate. Throughout the investigation, various spin states were examined, and the lowest spin state, predominantly a triplet state, remained during both the adsorption and the reaction process without spin crossing events. Gibbs free energy corrections (G_corr_) were calculated at 298.15 K and 1 atm. Additionally, to improve the accuracy of energy, single-point calculations were performed with the 6-311G(d,p) basis set for the Al, Si, O, C and H atoms and the RECP with LANL2DZ basis set for the Pd and Br atoms. The resulting free energies with free energy corrections (E_0_ + G_corr_) were reported for all reaction pathways. Cartesian coordinates of all the optimized structures are provided in the Supplementary information file. All calculations were performed with the Gaussian 09 program version D01^54^.

### Supplementary Information

Supplementary Information.

## Data Availability

All data generated or analysed during this study are included in this published article and its supplementary information files.
