# Supplementary material for: Investigation of the Suzuki–Miyaura cross-coupling reaction on a palladium H-beta zeolite with DFT calculations
Source: Sci Rep. 2024 Jan 5;14:611. doi: 10.1038/s41598-023-51116-x (PMC10770145; doi:10.1038/s41598-023-51116-x)
Supplement: Supplementary file 1 — Supplementary Information. [file 41598_2023_51116_MOESM1_ESM.docx]

**Investigation of the Suzuki-Miyaura Cross-Coupling Reaction on a Palladium H-Beta Zeolite with DFT Calculations**

Bundet Boekfa^1,2*^, Thana Maihom^1,2^, Masahiro Ehara^3^ and Jumras Limtrakul^4^

^1^ Division of Chemistry, Department of Physical and Material Sciences, Faculty of Liberal Arts and Science, Kasetsart University, Kamphaeng Saen Campus, Nakhon Pathom 73140, Thailand.

^2^Center for Advanced Studies in Nanotechnology for Chemical, Food and Agricultural Industries, Kasetsart University Institute for Advanced Studies, Kasetsart University,
Bangkok 10900, Thailand.
 ^3^Institute for Molecular Science, Nishigo-naka 38, Myodaiji, Okazaki 444-8585, Japan.

^4^ Department of Materials Science and Engineering, School of Molecular Science and Engineering, Vidyasirimedhi Institute of Science and Technology, Rayong, 21210 Thailand.

*email bundet.b@ku.ac.th

Optimized coordinate structures and their energies for the selected active region of Pd_4_-H-Beta zeolite and the probe molecules, with the imaginary frequency indicated for the transition state.

**01-AD1**

Al 10.048681 21.071407 21.262644

O 10.366713 20.915579 19.588597

O 10.781057 19.593596 21.782582

O 10.977667 22.413586 21.863758

Si 10.872633 23.489639 23.055514

Si 10.039883 21.713324 18.254547

Si 10.864408 18.646568 23.060673

O 8.422912 21.242153 21.709882

Si 6.877319 21.096312 21.309680

H 13.837637 19.113943 19.165221

Pd 13.024693 21.849828 20.882528

Pd 12.381021 19.327003 20.029228

Pd 14.170344 19.577014 21.903782

Pd 14.832661 20.505253 19.477079

C 14.269543 24.578727 20.890873

C 15.079201 24.183788 19.832252

C 15.124212 24.845593 18.616436

C 14.294069 25.954479 18.448177

C 13.473315 26.384599 19.487832

C 13.469923 25.707008 20.705479

H 14.297344 24.054948 21.846271

H 15.785581 24.512514 17.822338

H 14.297237 26.474209 17.494292

H 12.823459 27.246180 19.349283

H 12.854811 26.054474 21.529644

Br 16.297750 22.660408 20.116045

Optimized SCF Energy = -13964.4295 A.U. (6-31G(d,p)+LANL2DZ)

Sum of electronic and thermal Free Energies= -13966.199112A.U. (6-311G(d,p)+LANL2DZ)

**02-TS1**
Al 10.034559 21.079028 21.272948

O 10.377367 20.932118 19.598013

O 10.776782 19.597299 21.777980

O 10.970498 22.414658 21.849176

Si 10.876715 23.487727 23.046717

Si 10.038688 21.715884 18.255142

Si 10.856367 18.648340 23.056366

O 8.411736 21.221821 21.719788

Si 6.868763 21.094835 21.310152

H 13.705332 18.706811 19.082929

Pd 12.950561 21.784918 20.491492

Pd 12.251740 19.065563 19.901156

Pd 13.884239 19.555208 21.898940

Pd 14.718820 20.120714 19.463099

C 14.049673 24.351685 21.098805

C 14.122168 23.393188 20.079762

C 13.943489 23.707001 18.725063

C 13.550081 25.004636 18.405243

C 13.390594 25.958307 19.409626

C 13.629355 25.630766 20.747276

H 14.290374 24.093912 22.125065

H 14.102999 22.957338 17.956252

H 13.374507 25.264957 17.365689

H 13.068687 26.965033 19.151583

H 13.499907 26.376564 21.526770

Br 16.150219 22.103584 20.310738

Optimized SCF Energy = -13964.4248448 A.U. (6-31G(d,p)+LANL2DZ)

Sum of electronic and thermal Free Energies= -13966.194993 A.U. (6-311G(d,p)+LANL2DZ)
imaginary Frequency (6-31G(d,p)+LANL2DZ) -134.3208 cm^-1^

**03-IN1**

Al 10.051149 21.080541 21.247625

O 10.411310 20.931300 19.585760

O 10.800766 19.612468 21.792225

O 10.995767 22.397510 21.870132

Si 10.874330 23.495124 23.053178

Si 10.040970 21.719498 18.253691

Si 10.859983 18.647185 23.063155

O 8.425926 21.226172 21.695616

Si 6.874365 21.095594 21.308161

H 13.537144 18.617127 19.024578

Pd 13.138908 21.745027 21.212655

Pd 12.223741 19.061067 19.986001

Pd 14.083002 19.122494 21.863689

Pd 14.594947 20.056176 19.361767

C 13.699262 24.579809 20.986638

C 13.506691 23.425586 20.244966

C 13.545280 23.389460 18.861139

C 13.707212 24.601736 18.182965

C 13.852883 25.790615 18.893319

C 13.858176 25.780044 20.285708

H 13.713264 24.561747 22.072738

H 13.402882 22.458948 18.312504

H 13.698862 24.608201 17.095952

H 13.968008 26.727664 18.354459

H 13.984220 26.702605 20.846616

Br 15.781798 21.338677 21.356047

Optimized SCF Energy = -13964.44853 A.U. (6-31G(d,p)+LANL2DZ)

Sum of electronic and thermal Free Energies= -13966.219462 A.U. (6-311G(d,p)+LANL2DZ)

**04-AD2**
Al 10.109521 21.039739 21.258439

O 10.489597 20.979556 19.578143

O 10.968234 19.643163 21.819640

O 10.903197 22.431250 21.868099

Si 10.849390 23.486891 23.048008

Si 10.030360 21.721076 18.240980

Si 10.874709 18.639871 23.069911

O 8.453262 21.079742 21.631623

Si 6.886490 21.079816 21.306719

H 14.313488 21.332093 18.972595

Pd 13.726892 22.860992 21.788214

Pd 12.743491 21.627932 19.405409

Pd 13.203974 20.073564 21.523461

Pd 15.265003 22.708315 19.541739

C 13.744930 25.765379 22.409309

C 14.434470 24.720105 21.790171

C 15.674438 24.960761 21.220687

C 16.198717 26.249082 21.216975

C 15.526381 27.294620 21.835194

C 14.296069 27.047168 22.434181

H 12.782511 25.603944 22.871269

H 16.287092 24.156796 20.775200

H 17.110889 26.440035 20.674372

H 15.954333 28.294372 21.828600

H 13.739728 27.849383 22.915237

Br 15.719429 20.587002 21.264134

C 14.657216 24.205059 17.645294

C 15.825587 24.993515 17.686818

C 15.795626 26.272384 18.226710

C 14.606419 26.811508 18.709986

C 13.453929 26.035948 18.737129

C 13.487136 24.746931 18.220109

H 16.736832 24.592587 17.256224

H 16.703767 26.860792 18.305126

H 14.594504 27.817697 19.120517

H 12.539456 26.435249 19.166873

H 12.605698 24.118613 18.237070

O 15.847812 22.218414 16.688255

H 15.819170 21.327369 16.326491

O 13.398342 22.306477 16.588880

H 13.377500 21.382673 16.318856

B 14.629546 22.808728 16.933909

Optimized SCF Energy = -14372.6999845 A.U. (6-31G(d,p)+LANL2DZ)

Sum of electronic and thermal Free Energies= -14374.438488 A.U. (6-311G(d,p)+LANL2DZ)

**05-TS2**
Al 10.093383 21.104840 21.261747

O 10.381930 20.885779 19.575673

O 10.910147 19.727757 21.899750

O 10.932188 22.541342 21.754552

Si 10.872274 23.488418 23.056188

Si 10.036627 21.714947 18.256936

Si 10.874880 18.646015 23.076673

O 8.444806 21.191664 21.668746

Si 6.886915 21.091313 21.309672

H 13.718872 18.884033 19.639286

Pd 12.910584 23.737101 20.734476

Pd 12.314584 19.557019 19.499173

Pd 13.222452 20.632018 21.773696

Pd 13.957373 21.703647 19.397195

C 13.364079 26.278962 19.572424

C 14.083110 25.159900 19.999160

C 15.475461 25.147131 19.905896

C 16.122233 26.272443 19.383523

C 15.410366 27.393515 18.965624

C 14.026275 27.401913 19.072835

H 12.284371 26.298781 19.608209

H 16.053375 24.274753 20.209753

H 17.201335 26.272168 19.296260

H 15.941424 28.249351 18.554667

H 13.439524 28.264321 18.760964

Br 15.560763 21.712418 21.512591

C 14.678021 22.821447 17.707131

C 16.045464 22.756055 17.356435

C 16.568100 23.616636 16.402388

C 15.753574 24.603061 15.859786

C 14.455188 24.799808 16.329553

C 13.917064 23.916470 17.238257

H 16.637122 21.926973 17.732625

H 17.588500 23.488886 16.057734

H 16.129634 25.210072 15.045856

H 13.836693 25.590102 15.936229

H 12.863272 23.972525 17.494939

O 14.751902 20.210625 17.409700

H 15.466294 19.952701 16.819285

O 13.285456 21.656438 16.073476

H 12.918446 20.831291 15.745518

B 14.132598 21.412256 17.113074

Optimized SCF Energy = -14372.63208 A.U. (6-31G(d,p)+LANL2DZ)

Sum of electronic and thermal Free Energies= -14374.379765 A.U. (6-311G(d,p)+LANL2DZ)
imaginary Frequency (6-31G(d,p)+LANL2DZ) -207.2801 cm^-1^

**06-IN2**
Al 10.108755 21.079341 21.249315

O 10.471170 20.901959 19.561248

O 10.944542 19.710392 21.884920

O 10.923086 22.511757 21.772039

Si 10.880165 23.501530 23.050142

Si 10.034239 21.724020 18.252627

Si 10.872831 18.640543 23.078383

O 8.454764 21.143807 21.619343

Si 6.885309 21.087101 21.306214

H 13.945020 19.011080 19.601712

Pd 12.874468 22.722560 20.389976

Pd 12.553675 19.942538 19.161616

Pd 13.172143 20.368885 21.765793

Pd 15.097800 22.042710 18.967583

C 15.361704 24.352982 20.788398

C 14.626703 23.907296 19.666147

C 13.712441 24.800511 19.056397

C 13.521122 26.082457 19.591937

C 14.241850 26.497659 20.704425

C 15.167817 25.633348 21.296062

H 16.057189 23.677201 21.277588

H 13.204321 24.520125 18.136566

H 12.804267 26.754672 19.123164

H 14.083263 27.492089 21.113373

H 15.733802 25.946585 22.171308

Br 15.648815 20.873053 21.389216

C 14.779438 22.609396 17.082568

C 15.866791 22.913092 16.260748

C 15.676014 23.006115 14.878297

C 14.411886 22.820621 14.321274

C 13.325961 22.551475 15.151689

C 13.509035 22.453121 16.530944

H 16.856446 23.077750 16.679826

H 16.523806 23.241596 14.237559

H 14.272790 22.900929 13.246124

H 12.329002 22.416106 14.734370

H 12.663811 22.249637 17.179671

O 15.430900 19.830441 18.131260

H 15.897319 19.718009 17.286946

O 14.224489 17.739653 17.797734

H 14.882705 17.509230 17.134307

B 14.425990 18.939545 18.418863

Optimized SCF Energy = -14372.7167304 A.U. (6-31G(d,p)+LANL2DZ)

Sum of electronic and thermal Free Energies= -14374.455408 A.U. (6-311G(d,p)+LANL2DZ)

**07-TS3**
Al 10.076333 21.087424 21.261503

O 10.455664 20.921115 19.585267

O 10.792327 19.639904 21.831857

O 10.999780 22.471248 21.805018

Si 10.881127 23.499375 23.061582

Si 10.043038 21.708168 18.257206

Si 10.877444 18.643880 23.077575

O 8.437526 21.286193 21.640658

Si 6.880038 21.100266 21.307403

H 13.792275 18.410941 19.866916

Pd 12.727060 23.529322 20.177091

Pd 12.284707 19.275645 19.824293

Pd 13.167507 21.284131 21.741357

Pd 13.760619 21.353978 18.923652

C 13.627274 26.328913 20.070986

C 14.055810 25.050953 20.430574

C 15.104474 24.868799 21.333118

C 15.691076 25.991755 21.917222

C 15.257180 27.273546 21.581396

C 14.222273 27.441468 20.665330

H 12.836167 26.466886 19.336228

H 15.441870 23.865375 21.596191

H 16.495907 25.856708 22.633993

H 15.738258 28.139054 22.029571

H 13.873303 28.436707 20.397691

Br 15.557947 20.566330 21.346379

C 14.111458 23.736733 18.670667

C 15.328036 23.007148 18.627642

C 15.939397 22.778000 17.374981

C 15.433893 23.343982 16.216892

C 14.299831 24.161837 16.285513

C 13.645068 24.348174 17.492809

H 15.851186 22.720897 19.539847

H 16.835008 22.171304 17.335416

H 15.934728 23.176720 15.267010

H 13.914834 24.652284 15.395440

H 12.765159 24.974591 17.520032

O 14.676711 19.362870 18.011901

H 14.817558 19.336878 17.057060

O 13.547559 17.201665 17.994503

H 13.951389 17.049558 17.134445

B 13.845130 18.406710 18.574383

Optimized SCF Energy = -14372.6823527 A.U. (6-31G(d,p)+LANL2DZ)

Sum of electronic and thermal Free Energies= -14374.427183 A.U. (6-311G(d,p)+LANL2DZ)
imaginary Frequency (6-31G(d,p)+LANL2DZ) -239.9991 cm^-1^

**08-PR1**

Al 10.098792 21.082520 21.249293

O 10.459101 20.881164 19.565632

O 10.927302 19.708843 21.876032

O 10.903452 22.516641 21.778546

Si 10.872277 23.496382 23.057299

Si 10.040374 21.711345 18.260600

Si 10.880342 18.645810 23.076646

O 8.447924 21.156627 21.629705

Si 6.882937 21.087902 21.307037

H 13.762138 18.524315 19.691391

Pd 12.914177 23.355233 20.621217

Pd 12.323351 19.422758 19.393203

Pd 13.151894 20.600719 21.699283

Pd 14.162105 21.598498 19.078706

C 13.503632 25.737727 18.926888

C 14.258852 24.573576 19.217639

C 14.882782 24.476922 20.501654

C 14.698681 25.504956 21.450347

C 13.937020 26.621429 21.137090

C 13.352953 26.743155 19.865982

H 13.082584 25.836089 17.931873

H 15.595119 23.675783 20.698321

H 15.174581 25.408220 22.422348

H 13.790052 27.404735 21.875430

H 12.763576 27.622373 19.619741

Br 15.657288 20.980945 21.344078

C 14.624276 23.647346 18.106762

C 15.969870 23.580974 17.684074

C 16.320797 22.925263 16.516558

C 15.344484 22.283609 15.739688

C 14.016773 22.318751 16.134824

C 13.636833 23.005627 17.304002

H 16.715932 24.086784 18.284066

H 17.360846 22.913972 16.207332

H 15.627089 21.770515 14.823826

H 13.244021 21.824042 15.552038

H 12.588094 23.171016 17.518051

O 15.073912 19.507219 18.144013

H 15.521920 19.476934 17.288418

O 13.916561 17.388394 17.775281

H 14.530491 17.223583 17.052973

B 14.123911 18.565296 18.448259

Optimized SCF Energy = -14372.7542093 A.U. (6-31G(d,p)+LANL2DZ)

Sum of electronic and thermal Free Energies= -14374.494667 A.U. (6-311G(d,p)+LANL2DZ)

**14-AD4**
Al 10.094100 21.046472 21.237833

O 10.512800 20.972269 19.583491

O 10.901963 19.643958 21.827942

O 10.929164 22.460700 21.831930

Si 10.870422 23.500907 23.059204

Si 10.040180 21.712780 18.264636

Si 10.868762 18.642281 23.070006

O 8.449257 21.163548 21.641921

Si 6.884999 21.088353 21.306315

H 14.856919 20.328685 21.564419

Pd 12.888428 22.680252 20.645274

Pd 13.201596 20.007152 21.217830

Pd 15.048748 21.887081 22.211077

Pd 15.259006 22.089355 19.622984

C 13.726820 24.721075 19.310218

C 14.656312 23.974009 20.070237

C 15.330548 24.612670 21.132494

C 15.060705 25.937438 21.456639

C 14.122253 26.659372 20.714722

C 13.465161 26.056524 19.649225

H 13.255435 24.287906 18.427980

H 16.090080 24.070230 21.694749

H 15.587969 26.402241 22.286570

H 13.906873 27.693868 20.966810

H 12.736844 26.621690 19.071890

C 14.727162 21.311073 17.338253

C 15.916331 22.100999 17.311069

C 15.975789 23.371282 16.708579

C 14.846226 23.912498 16.122246

C 13.659742 23.166865 16.131419

C 13.608253 21.913823 16.723853

H 16.835084 21.655377 17.669628

H 16.913696 23.919881 16.713069

H 14.870863 24.903012 15.674785

H 12.755431 23.579778 15.687312

H 12.668601 21.387427 16.699283

O 15.866514 20.033081 19.077516

H 15.891186 19.322558 19.727265

O 13.393879 19.810748 18.993255

H 12.729198 20.462133 18.724635

O 14.772516 18.587666 17.518096

H 13.903770 18.183975 17.592332

B 14.693772 19.870311 18.157303
Optimized SCF Energy = -14435.3465901 A.U. (6-31G(d,p)+LANL2DZ)

Sum of electronic and thermal Free Energies= -14437.090953 A.U. (6-311G(d,p)+LANL2DZ)

**15-TS4**
Al 10.098226 21.059452 21.250723

O 10.497064 20.952999 19.586034

O 10.938871 19.650215 21.802771

O 10.949960 22.436751 21.851389

Si 10.850364 23.481445 23.041603

Si 10.038525 21.714807 18.273289

Si 10.883451 18.653467 23.053789

O 8.449542 21.155633 21.650755

Si 6.887505 21.086840 21.307185

H 14.784530 19.997166 20.857553

Pd 13.795048 22.898122 21.696807

Pd 12.976248 20.380837 20.708491

Pd 15.037848 20.634134 22.446593

Pd 15.253133 21.677451 19.935636

C 14.016003 25.654264 22.230094

C 14.781491 24.631024 21.669311

C 15.904101 24.914444 20.896050

C 16.188583 26.246457 20.593869

C 15.416619 27.279314 21.119964

C 14.338853 26.984822 21.947808

H 13.154050 25.435833 22.852728

H 16.517453 24.119487 20.471364

H 17.004170 26.474827 19.912234

H 15.660205 28.311621 20.879418

H 13.729907 27.778866 22.374208

C 14.672313 22.997894 18.386276

C 15.714352 23.658388 17.722603

C 15.598782 25.000470 17.403485

C 14.497495 25.730828 17.847027

C 13.484135 25.109607 18.567867

C 13.552626 23.737456 18.809006

H 16.586132 23.104317 17.383199

H 16.367825 25.476519 16.816793

H 14.435061 26.796134 17.638184

H 12.655869 25.695528 18.955379

H 12.727503 23.228860 19.309985

O 15.704070 20.557251 18.035106

H 16.456496 20.883226 17.526362

O 14.517636 21.636223 16.190061

H 14.446679 20.859120 15.625509

O 13.359655 20.628632 18.104326

H 12.571443 20.979787 17.672492

B 14.499671 21.232247 17.535876

Optimized SCF Energy = -14435.2981057 A.U. (6-31G(d,p)+LANL2DZ)

Sum of electronic and thermal Free Energies= -14437.042348 A.U. (6-311G(d,p)+LANL2DZ)
imaginary Frequency (6-31G(d,p)+LANL2DZ) -211.5838 cm^-1^

**16-IN4**
Al 10.122656 21.062504 21.235855

O 10.525250 20.969251 19.576499

O 10.974234 19.691488 21.858791

O 10.950179 22.495704 21.785470

Si 10.871648 23.493963 23.043730

Si 10.039112 21.710144 18.265148

Si 10.875021 18.646021 23.066631

O 8.467947 21.131592 21.635792

Si 6.897879 21.085869 21.307627

H 14.981795 20.662136 21.675459

Pd 12.853442 22.911500 20.242118

Pd 13.262186 20.351239 21.637797

Pd 14.921060 22.509104 21.991237

Pd 15.050964 21.108029 19.765039

C 13.547843 25.702540 19.400825

C 13.545312 24.817264 20.480768

C 14.148927 25.190662 21.683508

C 14.797872 26.427998 21.787717

C 14.816517 27.301376 20.705425

C 14.169941 26.945433 19.521930

H 13.103511 25.418382 18.448846

H 14.062728 24.574309 22.583004

H 15.264108 26.706761 22.730148

H 15.326122 28.257981 20.784141

H 14.169101 27.620986 18.668939

C 14.191673 22.788452 18.680806

C 15.410738 23.481490 18.497893

C 15.937467 23.623409 17.209491

C 15.270587 23.101441 16.105645

C 14.080225 22.392488 16.282860

C 13.556008 22.225091 17.557297

H 15.905168 23.953360 19.345407

H 16.861737 24.172418 17.069182

H 15.678833 23.244896 15.108360

H 13.557270 21.957063 15.434892

H 12.668081 21.621854 17.690859

O 15.546124 17.216595 17.789907

H 16.417225 17.515104 17.517148

O 15.107792 19.583914 18.015634

H 15.777172 19.746054 17.339708

O 13.618668 17.955819 18.888914

H 13.083910 18.735500 19.087951

B 14.765241 18.249925 18.232964

Optimized SCF Energy = -14435.3675074 A.U. (6-31G(d,p)+LANL2DZ)

Sum of electronic and thermal Free Energies= -14437.117938 A.U. (6-311G(d,p)+LANL2DZ)

**17-TS5**

Al 10.130581 21.069907 21.238457

O 10.526462 20.946481 19.570594

O 10.945718 19.680607 21.850327

O 10.980723 22.488433 21.783812

Si 10.875621 23.492308 23.039982

Si 10.039448 21.713535 18.269594

Si 10.875682 18.647784 23.066283

O 8.471321 21.164572 21.619886

Si 6.899530 21.089690 21.307312

H 15.101760 20.578681 21.638984

Pd 12.919882 22.791177 20.262980

Pd 13.363847 20.186916 21.522185

Pd 14.979400 22.382467 22.009962

Pd 15.183576 21.000911 19.737762

C 13.533297 25.588144 19.380647

C 13.582898 24.707988 20.462379

C 14.239675 25.084829 21.635042

C 14.899608 26.318283 21.703338

C 14.859587 27.190918 20.620218

C 14.152591 26.835252 19.471861

H 13.049516 25.299245 18.449698

H 14.192247 24.471953 22.540769

H 15.432410 26.589877 22.612601

H 15.372878 28.147152 20.672189

H 14.106824 27.511026 18.619793

C 14.251216 22.635241 18.698959

C 15.432053 23.376473 18.456976

C 15.924667 23.488216 17.153264

C 15.267153 22.881272 16.086977

C 14.125109 22.115921 16.319307

C 13.632521 21.977932 17.614203

H 15.922355 23.905255 19.272105

H 16.818879 24.076065 16.973059

H 15.649361 22.997860 15.076584

H 13.611047 21.620210 15.500197

H 12.740511 21.386345 17.793872

O 15.235076 17.659035 20.025301

H 16.054645 17.783089 19.539487

O 14.424507 18.778424 18.122368

H 13.712071 19.289581 17.721802

O 12.975092 18.214662 20.035188

H 12.205393 18.546674 19.555369

B 14.188703 18.246073 19.376207

Optimized SCF Energy = -14435.3485542 A.U. (6-31G(d,p)+LANL2DZ)

Sum of electronic and thermal Free Energies= -14437.095138 A.U. (6-311G(d,p)+LANL2DZ)
imaginary Frequency (6-31G(d,p)+LANL2DZ) -153.2001 cm^-1^

**18-PR2**

Al 10.091637 21.039523 21.250333

O 10.438683 20.935868 19.584137

O 10.896544 19.610530 21.794824

O 10.917171 22.460699 21.827689

Si 10.869382 23.496746 23.055727

Si 10.041003 21.709028 18.267929

Si 10.878911 18.644307 23.069938

O 8.447904 21.146638 21.681156

Si 6.889852 21.087192 21.310862

H 14.861415 20.109595 21.035124

Pd 12.953091 22.444769 20.677999

Pd 13.099889 19.825918 20.970628

Pd 14.836073 21.554338 22.294407

Pd 15.047411 21.178530 19.575093

C 13.814130 26.262136 19.486037

C 14.504399 25.059354 19.701873

C 15.013513 24.803902 20.988871

C 14.803532 25.704667 22.029390

C 14.102383 26.886143 21.799439

C 13.622624 27.167298 20.521131

H 13.439208 26.492264 18.491434

H 15.551603 23.870013 21.173136

H 15.177198 25.469662 23.021590

H 13.924879 27.584758 22.612608

H 13.081245 28.089475 20.329063

C 14.643913 24.059087 18.629799

C 15.763265 23.190829 18.575092

C 15.811088 22.165053 17.606148

C 14.738728 21.982699 16.712651

C 13.674137 22.874091 16.732854

C 13.641119 23.908210 17.668578

H 16.634598 23.392958 19.194885

H 16.717730 21.586669 17.468921

H 14.772952 21.165784 15.997903

H 12.847463 22.760760 16.037552

H 12.792084 24.580286 17.681201

O 15.699462 17.548296 19.701893

H 16.356906 18.009131 19.173310

O 14.299941 18.454191 18.033314

H 13.448319 18.875297 17.875885

O 13.400203 17.459400 20.093890

H 12.521929 17.513912 19.700679

B 14.444953 17.850821 19.265412

Optimized SCF Energy = -14435.431006 A.U. (6-31G(d,p)+LANL2DZ)

Sum of electronic and thermal Free Energies= -14437.173824 A.U. (6-311G(d,p)+LANL2DZ)

**00-Bromobenzene**

C 14.295343 24.600819 20.895071

C 15.109472 24.192537 19.847097

C 15.125479 24.849728 18.624261

C 14.293786 25.954164 18.452609

C 13.467929 26.384220 19.487336

C 13.470911 25.707319 20.703789

H 14.304961 24.067026 21.839141

H 15.772511 24.507307 17.824035

H 14.296429 26.477764 17.500679

H 12.822566 27.245932 19.345955

H 12.828587 26.037621 21.515363

Br 16.278470 22.632491 20.103586

Optimized SCF Energy = -244.773100351 A.U. (6-31G(d,p)+LANL2DZ)

Sum of electronic and thermal Free Energies= -244.762274 A.U. (6-311G(d,p)+LANL2DZ)

**00-Phenylboronic acid**
C 14.774944 22.599305 17.031439

C 15.852163 22.885251 16.179386

C 15.670890 23.013132 14.806473

C 14.400405 22.856325 14.256424

C 13.315792 22.571983 15.083489

C 13.504983 22.445593 16.455479

H 16.843845 23.007591 16.609274

H 16.517884 23.235011 14.162078

H 14.255578 22.955707 13.183527

H 12.324052 22.449447 14.655342

H 12.658503 22.223590 17.101529

O 16.252396 22.620687 19.059389

H 16.328319 22.523256 20.010265

O 13.882433 22.175976 19.337917

H 14.061521 22.097623 20.276645

B 14.982016 22.457014 18.565675

Optimized SCF Energy = -408.224580165 A.U. (6-31G(d,p))

Sum of electronic and thermal Free Energies= -408.22449 A.U. (6-311G(d,p))

**00-Phenylboronate anion**

C 14.740838 22.776737 17.096602

C 15.805915 23.022660 16.215588

C 15.651154 23.014175 14.830960

C 14.401556 22.749597 14.268797

C 13.323633 22.492970 15.114622

C 13.501631 22.506501 16.498313

H 16.789672 23.214551 16.646050

H 16.505598 23.208658 14.180099

H 14.272520 22.737736 13.186500

H 12.342465 22.275865 14.688067

H 12.662846 22.290236 17.160613

O 16.169900 22.091745 19.076563

H 16.568760 22.665328 19.735722

O 13.749536 22.266655 19.413815

H 14.096337 21.455816 19.790299

B 14.940543 22.840900 18.718644

O 15.084598 24.254250 19.158656

H 14.338344 24.357937 19.753234

Optimized SCF Energy = -484.081657353 A.U. (6-31G(d,p))

Sum of electronic and thermal Free Energies= -484.099409 A.U. (6-311G(d,p))

**00-Biphenyl**

C 13.773223 26.220044 19.502275

C 14.436168 25.006902 19.735960

C 14.968753 24.781856 21.013373

C 14.842946 25.732778 22.018940

C 14.182137 26.933379 21.770537

C 13.648030 27.172321 20.506689

H 13.376084 26.424553 18.510706

H 15.463366 23.836107 21.222341

H 15.255635 25.532515 23.004117

H 14.084060 27.677327 22.556086

H 13.138188 28.109452 20.299706

C 14.569638 23.997643 18.669858

C 15.702560 23.174940 18.592774

C 15.829473 22.224208 17.587421

C 14.824948 22.072265 16.634619

C 13.693078 22.881182 16.697729

C 13.567759 23.832547 17.703010

H 16.502720 23.305735 19.317581

H 16.720862 21.604235 17.541856

H 14.923863 21.328967 15.848585

H 12.899178 22.764825 15.964792

H 12.669120 24.442317 17.761201

Optimized SCF Energy = -463.254743430 A.U. (6-31G(d,p))

Sum of electronic and thermal Free Energies= -463.201371 A.U. (6-311G(d,p))

**Bare-Pd_4_-H-Beta 34T Pd_4_-H-Beta zeolite**

O 12.297494 18.828490 23.812810

O 10.746186 20.841798 17.105385

O 6.534795 21.086807 19.698403

O 10.746206 17.142405 22.503795

O 10.725205 24.962699 22.398693

O 12.301300 23.386601 23.811999

O 8.443905 21.763835 18.006606

O 10.623204 23.239191 18.200716

H 9.849899 18.824499 24.058000

H 9.832401 23.358498 24.067800

H 6.311402 19.889199 21.857999

H 6.263000 22.227898 21.926999

O 12.297500 19.155700 15.796400

Si 10.851108 19.351198 16.490106

Si 16.250100 18.476000 14.756800

Si 13.239500 18.480600 14.670200

O 14.743000 19.056700 14.809600

O 17.140100 19.179700 15.907600

Si 19.502500 21.054500 21.284900

O 19.196200 21.086800 19.698400

O 18.870300 19.713700 21.928400

Si 16.250100 19.508200 24.852400

Si 13.239503 19.503606 24.938994

O 14.743000 18.927500 24.799600

O 17.140100 18.804400 23.701600

Si 19.508200 21.734000 18.250900

O 18.804400 20.844100 17.100100

Si 18.632200 19.351900 16.503800

Si 18.632200 18.632200 23.105400

O 10.670499 18.266300 17.673900

O 18.842900 22.351900 21.986800

O 18.804400 17.140100 22.509100

O 16.220400 21.105300 24.608100

O 13.255999 21.106800 24.737000

O 18.870300 18.270500 17.680700

O 18.927500 23.241100 18.198000

Si 10.040498 16.251299 21.355202

Si 10.037600 16.927500 18.320800

Si 6.846803 21.733976 18.250896

Si 10.048598 24.743500 18.337691

O 10.348400 16.897100 19.906300

Si 19.508200 16.250100 21.358300

O 18.828500 24.958900 22.397900

Si 16.251300 22.701900 24.855600

Si 13.240599 22.710000 24.939301

Si 18.633000 23.512500 23.091600

O 14.744900 23.284500 24.802200

O 17.142400 23.407600 23.706900

Si 19.502500 16.929700 18.324300

Si 19.503600 24.744700 18.337500

O 19.196200 16.897400 19.910700

O 12.301300 27.259000 15.797200

Si 10.040500 28.912700 21.355200

Si 10.048596 25.902001 21.271504

O 10.623200 27.406300 21.408500

Si 10.037600 29.588900 18.320800

O 10.348400 29.558500 19.906300

O 10.693400 28.289700 17.618400

O 10.725199 25.682900 17.210499

Si 10.853600 27.130600 16.503800

O 10.401202 25.322801 19.804600

Si 16.251300 27.943700 14.753600

Si 13.240600 27.935600 14.669900

Si 18.633000 27.133100 16.517600

O 14.744900 27.361000 14.807000

O 17.142400 27.237900 15.902300

Si 19.508200 28.911500 21.358300

Si 19.503600 25.900900 21.271700

O 18.927500 27.404400 21.411100

Si 19.502500 29.591100 18.324300

O 19.196200 29.558800 19.910700

O 18.842900 28.293600 17.622400

O 18.828500 25.686700 17.211200

O 19.151100 25.322800 19.804600

H 20.961100 16.223100 21.580500

H 18.980000 14.879100 21.406300

H 18.902400 15.749400 17.685800

H 20.956100 16.919500 18.105900

H 19.616000 19.135400 15.433200

H 16.223100 17.023100 14.979000

H 16.838900 18.759800 13.440100

H 13.254500 17.022200 14.854000

H 12.713600 18.801300 13.335500

H 19.616000 18.848800 24.176000

H 16.838900 19.224400 26.169100

H 12.713600 19.182899 26.273700

H 8.588100 16.223200 21.580300

H 10.570500 14.881000 21.403700

H 10.634200 15.745700 17.681800

H 8.583500 16.919300 18.105600

H 9.795401 19.160200 15.485199

H 16.223200 29.396100 14.978700

H 16.838800 27.663600 13.435500

H 19.619900 27.297400 15.440600

H 18.927400 30.810700 17.738900

H 20.956100 29.580900 18.105900

H 20.961100 28.884500 21.580500

H 18.868000 29.721000 22.405100

H 8.583500 29.580700 18.105600

H 10.613300 30.806800 17.732300

H 13.254600 29.394000 14.853900

H 9.799200 27.276200 15.490000

H 12.713700 27.614800 13.335600

H 10.682500 29.723300 22.400000

H 8.588100 28.884600 21.580300

H 8.590200 25.915999 21.455400

H 8.590200 24.729600 18.153802

H 20.962000 25.915900 21.455500

H 19.619900 23.348200 24.168600

H 16.838800 22.982000 26.173700

H 12.713700 23.030800 26.273600

H 20.956200 21.064600 21.503200

H 20.961100 21.761100 18.028700

H 20.962000 24.729700 18.153700

H 6.318600 23.105000 18.202799

H 6.206700 20.924500 17.204100

Al 10.078657 21.067239 21.254318

O 10.428091 20.923624 19.575506

O 10.848154 19.622459 21.793052

O 10.983425 22.417407 21.875095

Si 10.864045 23.499649 23.057642

Si 10.040873 21.720754 18.247253

Si 10.857777 18.653935 23.048151

O 8.439327 21.222095 21.661684

Si 6.881396 21.094445 21.306699

H 14.146388 20.209998 19.229484

Pd 13.253984 21.812687 21.501752

Pd 12.612487 19.895770 19.853936

Pd 14.059450 19.353686 22.026581

Pd 15.381575 20.779403 20.265171

Optimized SCF Energy = -13719.6155311 A.U. (6-31G(d,p)+LANL2DZ)

Sum of electronic and thermal Free Energies= -13721.420947 A.U. (6-311G(d,p)+LANL2DZ)

**Full list of Gaussian 09, Rev. D.01**

Gaussian 09, Rev. D.01, M. J. Frisch, G. W. Trucks, H. B. Schlegel, G. E. Scuseria, M. A. Robb, J. R. Cheeseman, G. Scalmani, V. Barone, B. Mennucci, G. A. Petersson, H. Nakatsuji, M. Caricato, X. Li, H. P. Hratchian, A. F. Izmaylov, J. Bloino, G. Zheng, J. L. Sonnenberg, M. Hada, M. Ehara, K. Toyota, R. Fukuda, J. Hasegawa, M. Ishida, T. Nakajima, Y. Honda, O. Kitao, H. Nakai, T. Vreven, J. A. Montgomery, Jr., J. E. Peralta, F. Ogliaro, M. Bearpark, J. J. Heyd, E. Brothers, K. N. Kudin, V. N. Staroverov, R. Kobayashi, J. Normand, K. Raghavachari, A. Rendell, J. C. Burant, S. S. Iyengar, J. Tomasi, M. Cossi, N. Rega, J. M. Millam, M. Klene, J. E. Knox, J. B. Cross, V. Bakken, C. Adamo, J. Jaramillo, R. Gomperts, R. E. Stratmann, O. Yazyev, A. J. Austin, R. Cammi, C. Pomelli, J. W. Ochterski, R. L. Martin, K. Morokuma, V. G. Zakrzewski, G. A. Voth, P. Salvador, J. J. Dannenberg, S. Dapprich, A. D. Daniels, O. Farkas, J. B. Foresman, J. V. Ortiz, J. Cioslowski, and D. J. Fox, Gaussian, Inc., Wallingford CT, 2009.
